# Xuebijing Protects Against Septic Acute Liver Injury Based on Regulation of GSK-3β Pathway

**DOI:** 10.3389/fphar.2021.627716

**Published:** 2021-04-30

**Authors:** Liping Cao, Zhenghong Li, Yi Ren, Mengmeng Wang, Zhizhou Yang, Wei Zhang, Xiaoqin Han, Mengya Yao, Zhaorui Sun, Shinan Nie

**Affiliations:** ^1^ Nanjing University of Chinese Medicine, Nanjing, China; ^2^ Department of Emergency Medicine, Jinling Hospital, Medical School of Nanjing University, Nanjing, China; ^3^ Department of Nephrology, Jiangsu Province Hospital of Chinese Medicine, Affiliated Hospital of Nanjing University of Chinese Medicine, Nanjing, China

**Keywords:** Xuebijing, acute hepatic injury, GSK-3β, CREB, NF-κB

## Abstract

Xuebijing (XBJ), the only drug approved for the sepsis and multiple organ dysfunction, and its protective effects against acute liver injury (ALI) and its mechanism. The aim of this study was to evaluate the protective effect of XBJ on cecal ligation and perforation (CLP)-induced mouse ALI model and LPS-induced RAW264.7 cell ALI model. Mice were pretreated with XBJ before the CLP model was established, and serum and liver tissues were collected at the end of the experiment to assess the levels of inflammatory factors and liver injury. Results showed that XBJ pretreatment reduced liver/body weight, aspartate aminotransferase (AST) and alanine aminotransferase (ALT) activities in serum, and inhibited levels of pro-inflammatory factors in serum. Cells were treatment with XBJ and modeled by LPS modeling increased cell viability in the XBJ-treated group compared to the model group and XBJ also decreased serum pro-inflammatory factors in a dose-dependent manner. Western blot detected that XBJ also up-regulated the phosphorylated levels of glycogen synthase kinase-3β (p-GSK-3β) and cAMP-response element-binding protein (p-CREB) and down-regulated the phosphorylated level of nuclear factor kappa-B (p-NF-κB) in liver and cell. After overexpression of GSK-3β in cells, the mechanism was further investigated using CO-IP analysis. The binding of p-NF-κB and p-CREB to CREB-binding protein (CBP) was increased and decreased, respectively, indicating that GSK-3β regulated inflammation by regulating the binding of p-NF-κB and p-CREB to CBP. The present studies suggested that the hepatoprotective effect of XBJ may be through up-regulation of GSK-3β (Ser9) and increasing the binding of p-CREB to CBP, thereby alleviating the inflammatory response.

## Introduction

The incidence of sepsis is increasing yearly and is a common condition in intensive care unit (Fan et al., 2020). Systemic inflammatory responses and organ dysfunction are hallmarks of sepsis, and sepsis-induced mortality increases with multiple organ dysfunction (MODS), which is the leading cause of death in septic patients (Delano and Ward, 2016). The liver is one of the main organs for the removal of endotoxins and therefore patients with sepsis are vulnerable to liver injury (Lescot et al., 2012). Currently, the mechanism of acute liver injury (ALI) during sepsis is unknown. Some findings have suggested that the imbalance between pro-inflammatory and anti-inflammatory responses plays a crucial role in sepsis-induced ALI (Spapen, 2008; Castellheim et al., 2009; Lelubre and Vincent, 2018). Therefore, improving inflammation may be an important way to prevent and treat ALI-related diseases.

Glycogen synthase kinase 3 (GSK-3) is a serine/threonine protein kinase that freely phosphorylates and regulates a variety of signal-regulated proteins. GSK-3 plays a key role in regulating the production of pro-and anti-inflammatory cytokines (Xin et al., 2020). On the one hand, the effect of GSK-3 on inflammation is due to its ability to regulate the stability and activity of nuclear factor kappa-B (NF-κB), which is known to play an important role in the regulation of inflammation (Kotliarova et al., 2008). On the other hand, GSK3 regulates inflammatory responses by influencing the interaction of the transcription factor cAMP-response element-binding protein (CREB) with the CREB-binding protein (CBP) (Martin et al., 2005). Therefore, the search for drugs that modulate the GSK-3β signaling pathway is of great importance for the prevention and treatment of inflammation-related diseases.

XueBiJing (XBJ) is the only drug approved by Chinese National Medical Products Administration specifically for the treatment of sepsis and multiple organ dysfunction syndrome (Li et al., 2019). XBJ injection is an intravenous preparation made from five traditional Chinese medicines: roots of *Paeonia lactiflora* (Chishao), roots of *Angelica sinensis* (Danggui), rhizomes of *Ligusticum chuanxiong* (Chuanxiong), flowers of *Carthamus tinctorius* (Honghua) and roots of *Salvia miltiorrhiza* (Danshen) (Yin and Li, 2014). By reducing the release of inflammatory factors from macrophages, XBJ injections can reduce tissue damage caused by endotoxins and maintain vital organ physiological functions (Chen et al., 2018).

Based on these studies, we ventured to investigate the mechanism of action of GSK-3β and NF-κB with CREB signaling pathways in sepsis-induced ALI. Our study was based on CLP-induced ALI model in mice and LPS-induced inflammation model in RAW264.7 macrophages. We investigated the role of GSK-3β in the regulation of NF-κB and CREB signaling pathways during sepsis-induced ALI. The results indicated that XBJ inhibited the production of pro-inflammatory factors, including tumor necrosis factor-α (TNF-α), interleukin-6 (IL-6), interleukin-12 (IL-12) and interleukin-1β (IL-1β). It also promoted the production of the anti-inflammatory factor interleukin-10 (IL-10) by up-regulating the expression level of GSK-3β, which inhibiting the expression of NF-κB and activating the CREB signaling and transmission pathway. These findings provide a possible mechanism for XBJ on the protection of the liver against ALI in sepsis.

## Materials and Methods

### Reagents and Antibodies

Xuebijing (XBJ) was purchased from Tianjin Hongri Pharmaceutical Co. (Tianjin, China). Dexamethasone (DXM) Sodium Phosphate Injection was purchased from Jilin Aodong Pharmaceutical Industry Group Yanji Co., Ltd. Mouse IL-10 ELISA kit (ab255729, Abcam), Mouse TNF-α ELISA kit (ab208348, Abcam), Mouse IL-6 ELISA kit (ab100713, Abcam), Mouse IL-12 p40 ELISA kits (ab236717, Abcam), Mouse IL-1β ELISA kits (ab197742, Abcam) were used to detect inflammatory factor levels in serum. Anti-GSK-3β (phospho S9) (ab75814, Abcam), GSK-3β (9832, CST), p-NF-κB (8242, CST), NF-κB (abs131170, absin), CREB (Ser133) (9198, CST) and CREB (9104, CST) were used as the primary antibody, and Goat anti-Rabbit (AlexaFluor^®^488) (ab150077, Abcam) was used as the second antibody.

### Animals and Treatments

C57BL/6 mice (18–22 g) were purchased from Hangzhou Ziyuan Experimental Animal Technology Co. LTD. The mice were housed in a laboratory animal care facility at a constant temperature 22 ± 2°C and humidity 50 ± 10%, with a 12/12 h light/dark cycle.

In the present study, mice were suffered from cecal ligation and perforation (CLP) surgery to induce septic ALI as previously reported (Chen et al., 2017). After 1 week of respite, C57BL/6 mice were randomly divided into five groups (*n* = 18): Con: control group that underwent sham surgery and distilled water gavage; CLP: model group with CLP surgery and distilled water gavage; L-XBJ: group with CLP surgery + low dose of XBJ group (4 ml/kg/day XBJ intraperitoneal injection); H-XBJ: group with CLP surgery + high dose of XBJ group (8 ml/kg/day XBJ intraperitoneal injection); DXM: group with CLP surgery +5 mg/kg/day DXM intraperitoneal injection. The XBJ intervention experiment lasted for 5 days, during which the mice had free access to food and water. Mice in all groups received CLP surgery on day 6 after the end of all interventions, except for the mice in the control group which received sham surgery, and the CLP method was referred to previous study (Chen et al., 2017). After the end of CLP surgery, mice in each group were injected intraperitoneally at 0, 2 and 12 h at the corresponding concentrations of XBJ or DXM dosing concentration, respectively. On day 7, mice were sacrificed to obtain liver tissue and serum.

### Histological Analysis

In order to observe histological changes, liver tissues were fixed with 4% paraformaldehyde, embedded in paraffin and cut into sections of 4–5 μm thickness. After removal of paraffin and dehydration, the sections were stained with hematoxylin and eosin (H and E), and pathological changes in liver tissue were examined under an optical microscope.

### Immunofluorescence Assay

Immunofluorescence was used to assess GSK-3β (Ser9) in tissues and cells. Cells were fixed with 4% paraformaldehyde for 20 min and permeabilized with 0.5% Triton X-100 for 20 min. Tissues and cells were blocked with 5% BSA blocking solution for 60 min at room temperature, followed by washing with PBS. Samples were then incubated with GSK-3β (Ser9) antibody (ab75814, abcam) overnight at 4°C and further stained with Goat anti-Rabbit (AlexaFluor^®^488) (ab150077, abcam) secondary antibody. Afterwards, the samples were stained with 4’,6-diamidino-2-phenylindole (DAPI, C0060, Solarbio, China) and observed with a fluorescence microscope (BX63, Olympus, Japan) to obtain representative fluorescence images.

### Biochemical Indicators of Liver Function

The alanine aminotransferase (ALT) (C009-2-1), aspartate aminotransferase (AST) (C010-2-1) and myeloperoxidase (MPO) (A044-1-1) assay kits from Nanjing Jianjian Bioengineering Institute were used here to concrete operation according to the manufacturer’s instructions.

### Enzyme Linked Immunosorbent Assay (ELISA)

The levels of IL-10, TNF-α, IL-6, IL-12 and IL-1β in serum and supernatant were analyzed by the ELISA kits following the manufacturer’s instructions, respectively.

### Western Blot Analysis

For protein blot analysis, protein extracts were separated using 12% SDS-PAGE gel electrophoresis and electroblotted onto polyvinylidene difluoride membranes. After confinement with PBST containing 5% bovine serum albumin, the membranes were incubated with primary antibodies (1:1,000) overnight at 4°C and then incubated with HRP-coupled secondary antibodies for 2 h at room temperature. The immune blot bands were visualized *via* an ECL luminescence reagent (K002, Affinity) and quantified by ImageJ.

### Cell Culture and Administration

RAW264.7 cells were cultured in Dulbecco’s modified Eagle’s medium (DMEM, Invitrogen, United States) supplemented with 10% fetal bovine serum (FBS, Gibco, United States) containing double antibiotics (100 μg/ml of penicillin and 100 μg/ml of streptomycin, Beyotime, China) at 37°C with 5% CO_2_. Cells were divided into five groups: Control group without LPS treatment; LPS model group was treated with 1 μg/ml lipopolysaccharide (LPS); 100-XBJ, group was treated with 1 μg/ml LPS and a 100-fold dilution of XBJ; 50-XBJ, group was treated with 1 μg/ml LPS and a 50-fold dilution of XBJ; 25-XBJ was treated with 1 μg/ml LPS and a 25-fold dilution of XBJ. Different concentrations of drugs were administered for 2 h prior to model, and normal medium was given to the Con group, medium containing 1 μg/ml LPS was given to the LPS model and XBJ treatment group. Incubation was continued for 24 h, followed by subsequent experiments.

### Detection of Cell Viability

The viability of RAW264.7 cells treated with different conditions was analyzed by 3-(4,5-dimethyl2-thiazolyl)-2,5-diphenyl-2-H-tetrazolium bromide (MTT, Solarbio, China) assay (Song et al., 2018). The absorbance (OD 490 nm) was recorded by a microplate reader (Nanodrop, Thermo, United States).

### Co-IP Analysis

RAW264.7 cells were lyzed with IP lysis buffer (10 mM Tris pH 7.4, 25 mM NaCl2, 5 mM EDTA, 0.1% NP40, 1% protease inhibitor). Cell lysates were pre-cleared with Protein A magnetic beads (73778, CST) and then mixed with CBP (7389, CST) or control IgG (B900620, Proteintech) for 1 h at 4°C. Immunoprecipitates were captured with Protein A magnetic beads and analyzed by Western blotting with antibodies against p-NF-κB (8242, CST) or CREB (ser133) (9198, CST), respectively.

### Data Analysis

All data were expressed as mean ± SD (standard deviation). One-way analysis of variance (ANOVA) were utilized to determine statistical significance. Compared with the control group, #*p* < 0.05, ##*p* < 0.01 were considered to be significant; Compared with the CLP group, *p < 0.05, **p < 0.01 were considered to be significant. Statistical analysis was performed with GraphPad Prism 8.

## Results

### Xuebijing Alleviates Acute Liver Injury in C57BL/6 Mice With Cecal Ligation and Perforation-Induced Sepsis

**FIGURE 1:**
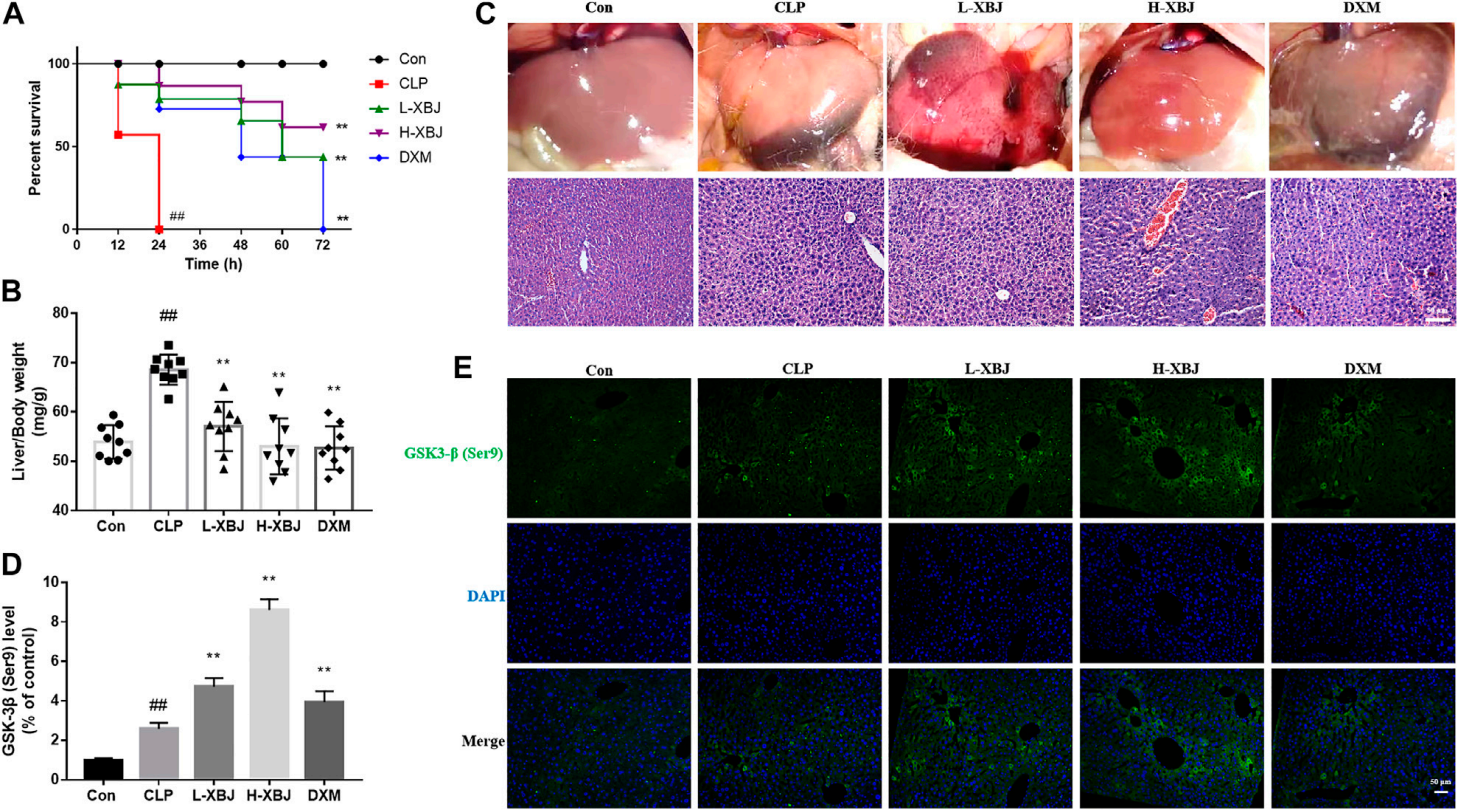
XBJ alleviates acute liver injury (ALI) in C57BL/6 mice with cecal ligation perforation (CLP) sepsis **(A)** XBJ improved survival in mice with ALI from sepsis. **(B)** XBJ reduced liver/body weight in mice with ALI from sepsis **(C)** Phenotype and H and E staining of livers from each group of ALI mice. **(D)** Representative images of GSK-3β immunofluorescence *in vivo* **(E)** The expression of GSK-3β in liver of mice with ALI. Con, control group that underwent sham surgery; CLP, model group with CLP surgery; L-XBJ: group with CLP surgery + XBJ low dose group (4 ml/kg/day); H-XBJ: group with CLP surgery + XBJ high dose group (8 ml/kg/day); DXM: group with CLP surgery + Dexamethasone (5 mg/kg/day). Compared with the control group, #*p* < 0.05, ##*p* < 0.01 were considered to be significant; Compared with the CLP group, *p < 0.05, **p < 0.01 were considered to be significant. Data are expressed as mean ± SD.

XBJ treatment significantly improved survival rate and reduced liver/body weight in septic ALI mice (*p* < 0.01, Figures 1A,B). As shown in Figure 1C, the liver phenotype indicated that the results in the XBJ-treated group were similar to those in the control group. Usually, liver injury is accompanied by inflammatory infiltration, in order to further evaluate the protective effects of XBJ on CLP-induced ALI mice, pathological examination of liver tissues was performed, and inflammatory cell infiltration in the liver tissue of the CLP group compared to the control group, whereas in the L-XBJ and H-XBJ groups, inflammatory cell infiltration was reduced. It could be seen that the fluorescence intensity of GSK-3β (Ser9) was significantly higher in the CLP group than in the control group and was stronger in L-XBJ and H-XBJ groups (Figures 1D,E).

### Xuebijing Improves Functions of Liver

**FIGURE 2:**
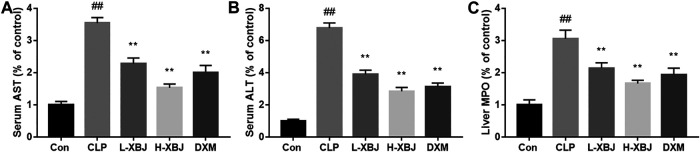
XBJ improves liver function **(A)** XBJ reduced AST in serum of mice with ALI induced by CLP. **(B)** XBJ reduced ALT in serum of mice with ALI induced by CLP **(C)** XBJ reduces MPO in the liver of mice with CLP-induced ALI. Con: control group that underwent sham surgery; CLP: model group with CLP surgery; L-XBJ: group with CLP surgery + XBJ low dose group (4 ml/kg/day); H-XBJ: group with CLP surgery + XBJ high dose group (8 ml/kg/day); DXM: group with CLP surgery + Dexamethasone (5 mg/kg/day). Compared with the control group, #*p* < 0.05, ##*p* < 0.01 were considered to be significant; Compared with the CLP group, *p < 0.05,**p < 0.01 were considered to be significant. Data are expressed as mean ± SD.

In liver function tests, AST is an indicator of hepatocyte damage, while AST is a criterion for hepatocyte necrosis. When the liver is inflamed, ALT is released from hepatocytes into bloodstream, so serum aminotransferase number is an important indicator of the degree of liver disease. As shown in Figures 2A,B, the AST and ALT activities in serum of the CLP model group were significantly higher than those of the control group (*p* < 0.01). Compared with the CLP model group, the L-XBJ and H-XBJ groups also showed significantly lower serum ALT and AST activities after XBJ pretreatment (*p* < 0.01). This indicated that XBJ could significantly reduce the degree of liver injury. MPO is a neutrophil-specific enzyme that can be measured in liver tissue to determine the extent of neutrophil infiltration. At the same time, the MPO activity of L-XBJ and H-XBJ was also significantly reduced compared to the CLP model group, which indicated that XBJ reduced neutrophil infiltration in septic ALI mice (*p* < 0.01, Figure 2C).

### Xuebijing Modulates the Levels of Inflammatory Factors in Serum of Cecal Ligation and Perforation-Induced Acute Liver Injury Mice

**FIGURE 3:**
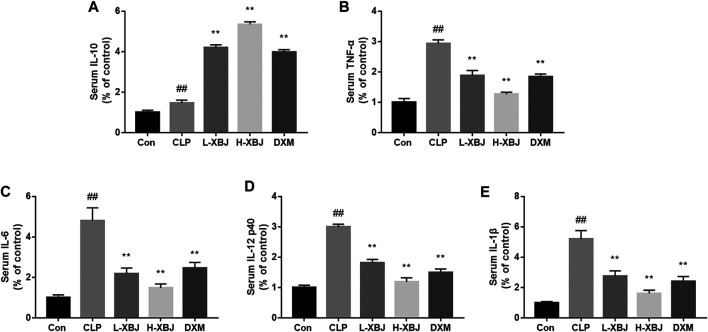
XBJ modulates the serum levels of inflammatory factors in mice with CLP-induced ALI **(A)** XBJ increased IL-10 in serum of mice with CLP-induced ALI. **(B)** XBJ decreased TNF-α in serum of mice with CLP-induced ALI **(C)** XBJ decreased IL-6 in serum of mice with CLP-induced ALI. **(D)** XBJ decreased IL-12 in serum of mice with CLP-induced ALI **(E)** XBJ decreased IL-1β in serum of mice with CLP-induced ALI. Con: control group that underwent sham surgery; CLP: model group with CLP surgery; L-XBJ: group with CLP surgery + XBJ low dose group (4 ml/kg/day); H-XBJ: group with CLP surgery + XBJ high dose group (8 ml/kg/day); DXM: group with CLP surgery + Dexamethasone (5 mg/kg/day). Compared with the control group, #*p* < 0.05, ##*p* < 0.01 were considered to be significant; Compared with the CLP group, *p < 0.05, **p < 0.01 were considered to be significant. Data are expressed as mean ± SD.

IL-6, IL-12, IL-1β and TNF-α are important inflammatory cytokines in sepsis. Compared with the control group, IL-6, IL-12, IL-1β and TNF-α were significantly increased in CLP model group (*p* < 0.01) and decreased in the L-XBJ and H-XBJ groups (*p* < 0.01, Figures 3B–E). IL-10 inhibits the production of pro-inflammatory cytokines including TNF-α, IL-1, and IL-6, acting as a suppressor of the inflammatory response. In Figure 3A, the IL-10 in the L-XBJ and H-XBJ groups was increased significantly than in the CLP model group. Both results suggested that XBJ could promote the production of anti-inflammatory factor IL-10 and inhibit the production of pro-inflammatory factor *in vivo*.

### Xuebijing Regulates the Activity of GSK-3β, NF-κB and CREB in the Liver of Cecal Ligation and Perforation-Induced Acute Liver Injury Mice

**FIGURE 4:**
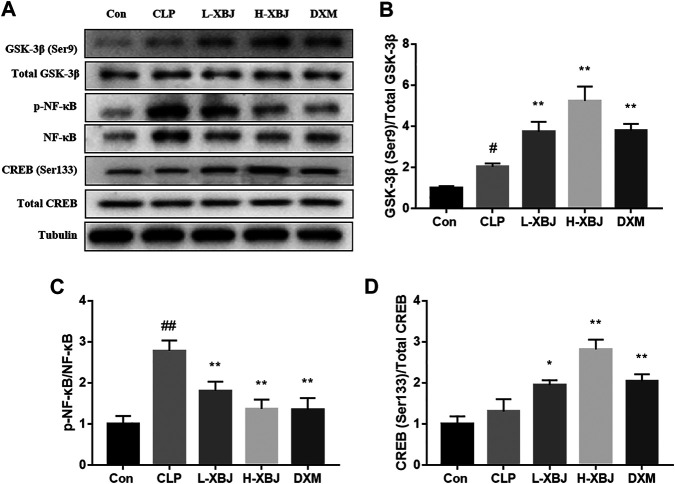
XBJ regulates the activities of GSK-3β, NF-κB and CREB in the liver of CLP-induced ALI mice **(A)** Representative immunoblots of GSK-3β, NF-κB and CREB protein expression in liver lysates. **(B-D)** Administration of XBJ increased GSK-3β and CREB activity, while NF-κB activity was reduced *in vivo.* Con: control group that underwent sham surgery; CLP: model group with CLP surgery; L-XBJ: group with CLP surgery + XBJ low dose group (4 ml/kg/day); H-XBJ: group with CLP surgery + XBJ high dose group (8 ml/kg/day); DXM: group with CLP surgery + Dexamethasone (5 mg/kg/day). Compared with the control group, #*p* < 0.05, ##*p* < 0.01 were considered to be significant; Compared with the CLP group, *p < 0.05, **p < 0.01 were considered to be significant. Data are expressed as mean ± SD.

The expressions of GSK-3β (Ser9), GSK-3β, p-NF-κB, NF-κB, p-CREB and CREB in liver tissues were measured by western blot (Figure 4A). Compared with the control group, the phosphorylation of Ser9 on GSK-3β and phosphorylation of Ser133 on CREB were both increased in the CLP model group, and this increasing trend was more pronounced in the L-XBJ and H-XBJ groups (Figures 4B,D). P-NF-κB/NF-κB was increased in the CLP model group compared to the control group, however, this increasing trend was reversed in the L-XBJ and H-XBJ groups (Figure 4C).

### Xuebijing Treatment Increases the Viability and GSK-3β (Ser9) Expression of RAW264.7 Cells

**FIGURE 5:**
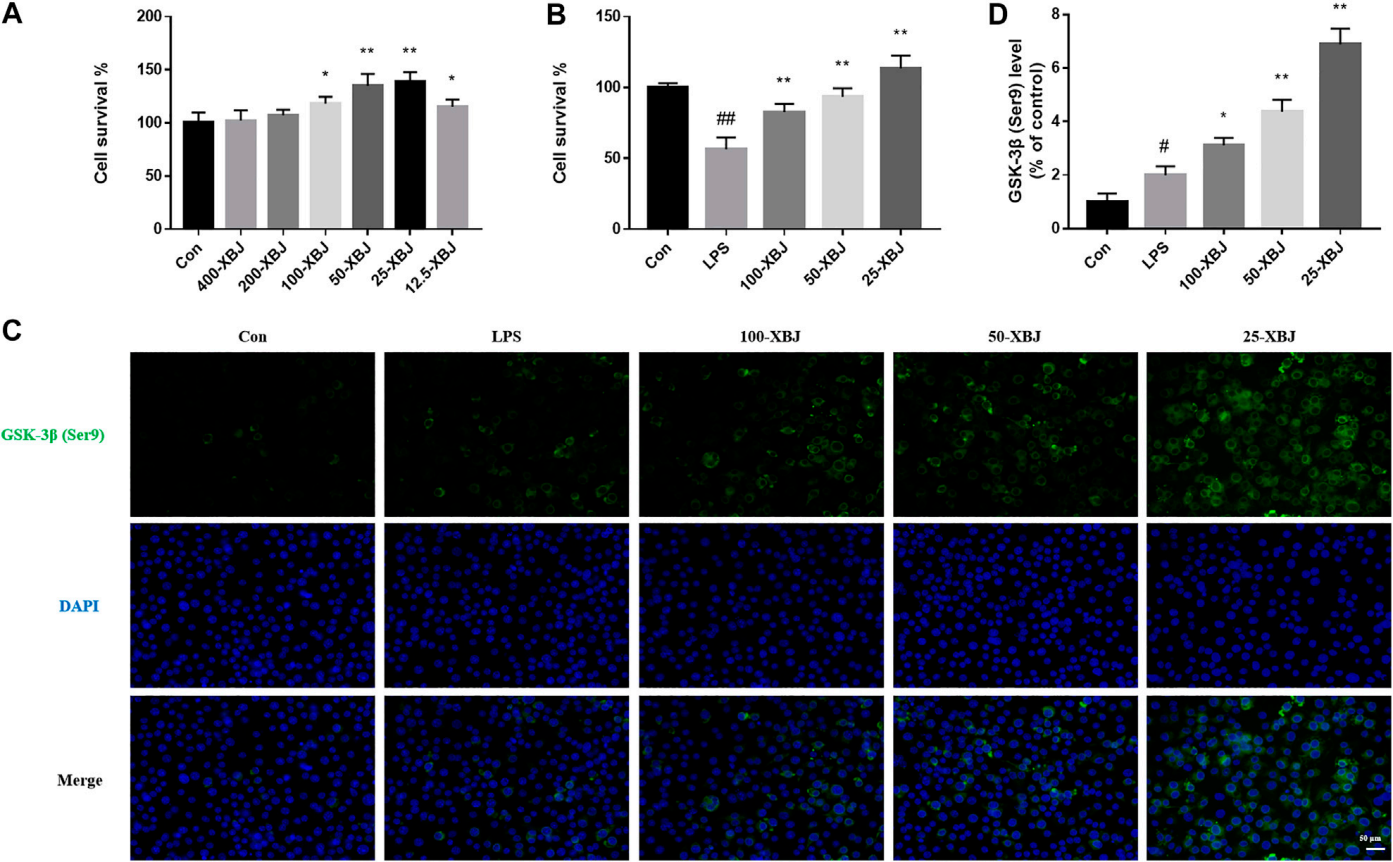
XBJ treatment increases the viability and GSK-3β (Ser9) expression of RAW264.7 cells **(A)** Concentration screening for XBJ cell experiments. **(B)** XBJ improved the viability of LPS-induced sepsis ALI cells **(C)** Representative images of GSK-3β immunofluorescence *in vitro*. **(D)** The expression of GSK-3β in cell with ALI. Con: control group without LPS treatment; LPS: model group with LPS treatment; 100-XBJ: group with LPS treatment + XBJ injection diluted 100 times; 50-XBJ: group with LPS treatment + XBJ injection diluted 50 times; 25-XBJ: group with LPS treatment + XBJ injection diluted 25 times. Compared with the control group, #*p* < 0.05, ##*p* < 0.01 were considered to be significant; Compared with the CLP group, *p < 0.05, **p < 0.01 were considered to be significant. Data are expressed as mean ± SD.

RAW264.7 cells were treated with different dilutions of XBJ to test the viability of the cells after exposure to XBJ and to determine the optimal concentration of XBJ for use in subsequent experiments. XBJ increased the viability of RAW264.7 cells in a concentration-dependent manner, with a significant increase in cell viability at a dilution of 25 (*p* < 0.05, Figure 5A). After LPS treatment, there was a concentration-dependent increase in the viability of RAW264.7 cells (Figure 5B). In Figures 5C,D, it can be seen that the fluorescence intensity of GSK-3β (Ser9) was significantly higher in the LPS group than in the control group and was stronger in the 100-XBJ, 50-XBJ and 25-XBJ groups.

### Xuebijing Modulates the Levels of Inflammatory Factors in Lipopolysaccharide-Induced Acute Liver Injury Cells

**FIGURE 6:**
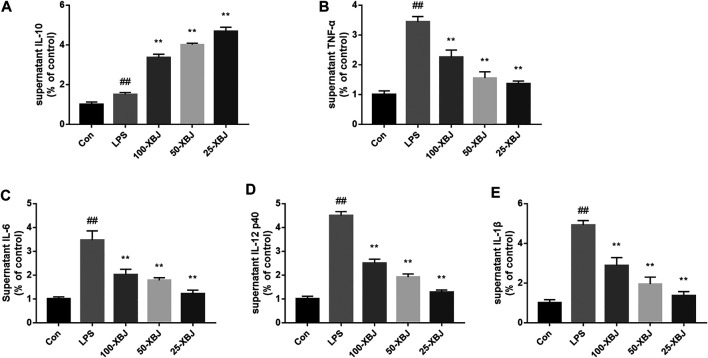
XBJ modulates the levels of inflammatory factors in LPS-induced ALI cells **(A)** XBJ increased IL-10 in RAW264.7 cells with LPS-induced ALI. **(B)** XBJ decreased TNF-α in RAW264.7 cells with LPS-induced ALI **(C)** XBJ decreased IL-6 in RAW264.7 cells with LPS-induced ALI. **(D)** XBJ decreased IL-12 in RAW264.7 cells with LPS-induced ALI. **(E)** XBJ decreased IL-1β in RAW264.7 cells with LPS-induced ALI. Con: control group without LPS treatment; LPS: model group with LPS treatment; 100-XBJ: group with LPS treatment + XBJ injection diluted 100 times; 50-XBJ: group with LPS treatment + XBJ injection diluted 50 times; 25-XBJ: group with LPS treatment + XBJ injection diluted 25 times. Compared with the control group, #*p* < 0.05, ##*p* < 0.01 were considered to be significant; Compared with the CLP group, *p < 0.05, **p < 0.01 were considered to be significant. Data are expressed as mean ± SD.

IL-6, IL-12, IL-1β and TNF-α were significantly increased in LPS model group (*p* < 0.01) and decreased in the 100-XBJ, 50-XBJ and 25-XBJ groups compared to the control group (*p* < 0.01, Figures 6B–E). In Figure 6A, the IL-10 in the 100-XBJ, 50-XBJ and 25-XBJ groups was increased significantly than that in the control and LPS model groups. Two results suggested that XBJ could promote the anti-inflammatory factor IL-10 and inhibit the pro-inflammatory factors *in vitro*.

### Xuebijing Regulates the Activities of GSK-3β, NF-κB and cAMP-Response Element-Binding Protein in Lipopolysaccharide-Induced Acute Liver Injury Cells

**FIGURE 7:**
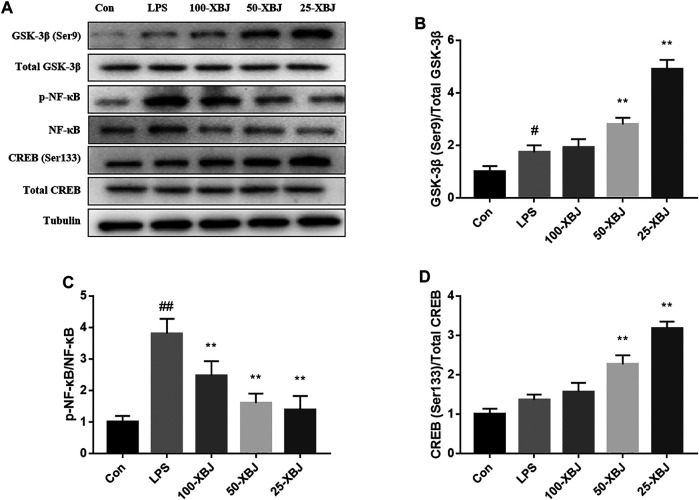
XBJ regulates the activities of GSK-3β, NF-κB and CREB in LPS-induced ALI cells **(A)** Representative immunoblots of GSK-3β, NF-κB and CREB protein expression in cell lysates. **(B-D)** Administration of XBJ increased GSK-3β and CREB activity, while NF-κB activity was reduced *in vitro.* Con: control group without LPS treatment; LPS: model group with LPS treatment; 100-XBJ: group with LPS treatment + XBJ injection diluted 100 times; 50-XBJ: group with LPS treatment + XBJ injection diluted 50 times; 25-XBJ: group with LPS treatment + XBJ injection diluted 25 times. Compared with the control group, #*p* < 0.05, ##*p* < 0.01 were considered to be significant; Compared with the CLP group, *p < 0.05, **p < 0.01 were considered to be significant. Data are expressed as mean ± SD.

The expressions of GSK-3β (Ser9), GSK-3β, p-NF-κB, NF-κB, CREB (Ser133) and CREB in cells were measured by western blot (Figure 7A). Compared withe the control group, the phosphorylation of Ser9 on GSK-3β and phosphorylation of Ser133 on CREB were both increased in the LPS model group, and this increasing trend was more pronounced in the 100-XBJ, 50-XBJ and 25-XBJ groups (Figures 7B,D). Compared withe the control group, p-NF-κB/NF-κB was increased in the LPS model group, however, this increasing trend was reversed in the 100-XBJ, 50-XBJ, and 25-XBJ groups (Figure 7C).

### Overexpression of GSK-3β Regulates the Binding of CREB-Binding Protein to p-NF-κB and p-CREB

**FIGURE 8:**
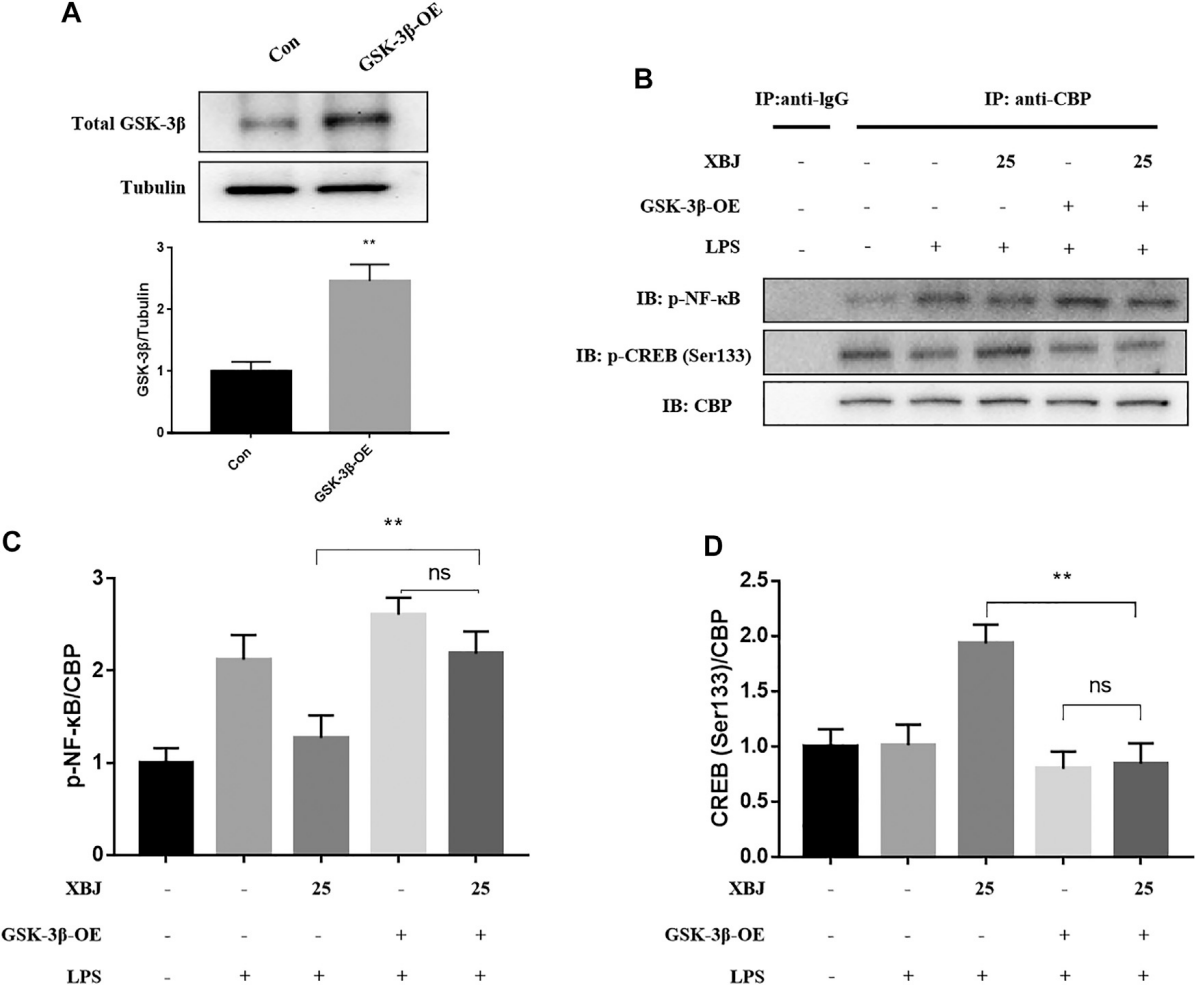
Overxepression of GSK-3β regulates the expression pNF-κB and CREB (Ser133) **(A)** GSK-3β was successfully overexpressed in cells. **(B)** Representative immunoblots of p-NF-κB and p-CREB **(C)** GSK-3β-OE increased the expression of pNF-κB in cells. **(D)** GSK-3β-OE decreased the expression of CREB in cells. Con: control group; GSK-3β-OE: GSK-3β overexpression group. *p < 0.05, **p < 0.01 were considered to be significant. Data are expressed as mean ± SD.

In addition, to confirm whether GSK-3β is effective in reducing inflammation levels, RAW264.7 cells were transfected using lentiviral vectors overexpressing GSK-3β (Le^GSK−3β^, Genechem, China) and negative control vectors (Le^Ctrl^, Genechem, China). Figure 8A showed that GSK-3β was successfully overexpressed. After LPS modeling, p-NF-κB/CBP was significantly higher in the Le^GSK−3β^ + XBJ group than in the Le^Ctrl^ + XBJ group, while p-CREB/CBP was significantly lower in the Le^GSK−3β^ + XBJ group than in the Le^Ctrl^ + XBJ group, while the binding of CBP and p-NF-κB or p-CREB with the addition or non-addition of XBJ did not change significantly (Figures 8B–D). The results showed that the XBJ regulates the binding of CBP and p-NF-κB or p-CREB by affecting GSK-3β.

## Discussion

Sepsis can be caused by infection, burns or trauma (Protzer et al., 2012). The liver is the primary target of inflammatory factor-mediated inflammatory responses, manifesting as sepsis-induced inflammation and injury (Wang et al., 2014). Hepatic organ dysfunction is an early symptom of sepsis, and the development of liver failure can lead directly to increased mortality (Poeze et al., 2008). In China, XBJ is used as a treatment for sepsis and organ dysfunction syndrome. Studies had shown that XBJ could inhibit the uncontrolled release of inflammatory mediators, alleviate excessive innate immune responses and maintain physiological function of organs (Qi et al., 2011; Wang et al., 2015; Hong et al., 2020). These findings suggest that treating sepsis with XBJ is an effective strategy, however, few studies have investigated the specific protective mechanisms of XBJ against sepsis-induced liver injury.

In mammals, GSK3 exists in two major subtypes, GSK-3α and GSK-3β (Woodgett, 1990). GSK-3 is now known to target a variety of substrates ranging from transcription factors such as NF-κB and CREB (Jope and Johnson, 2004). NF-κB regulates many different cellular processes, including responses to pro-inflammatory cytokines (Hoeflich et al., 2000). NF-κB regulation can be phosphorylation-dependent with cellular coactivators to mediate, including CBP (Ghosh et al., 1998). It has been reported that GSK-3β inhibition enhances the binding of CREB (Ser133) and inhibits the binding of NF-κB p65 (Ser276) to the nuclear coactivator CBP (Martin et al., 2005). CREB is known to activate IL-10 production by binding to the IL-10 promoter (Hu et al., 2006), and the ability of CREB activity correlates with the production of pro- and anti-inflammatory cytokines (Martin et al., 2005).

*In vivo* experiments based on mouse survival, HE staining, serum AST and ALT measurements and inflammatory factor assay showed that XBJ was effective in reducing sepsis-induced liver injury (Figure 1). The pro-inflammatory cytokines TNF-α, IL-6, IL-12 and IL-1β exacerbated inflammation, while the anti-inflammatory cytokine IL-10 inhibited pro-inflammatory cytokines to maintain a steady state (Bogacz et al., 2020). TNF-α, IL-6, IL-12 and IL-1β play an important role in sepsis, and levels of TNF-α are negatively correlated with survival in septic patients (Baghel et al., 2014). In contrast, XBJ decreased the levels of pro-inflammatory cytokines TNF-α, IL-6, IL-12 and IL-1β and increasing the levels of anti-inflammatory cytokine IL-10 in mice and cell, which is consistent with previous findings (Li et al., 2016; Li et al., 2021). In the present study, XBJ treatment resulted in phosphorylation of Ser9 on GSK-3β (the inactive form of GSK-3β), accompanied by the inhibition of NF-κB activity and the enhancement of CREB activity, suggesting that the inhibition of GSK-3β by XBJ alleviated the inflammatory response, in agreement with previous findings (Martin et al., 2005; Hu et al., 2006). Further GSK-3β overexpression CO-IP experiments showed that XBJ promoting CREB activity and the anti-inflammatory cytokine IL-10 and inhibiting NF-κB activity and pro-inflammatory cytokines.

In conclusion, this study shows that XBJ treatment is not only effective in reducing CLP-induced liver injury *in vivo*, but also in improving RAW264.7 cell viability *in vitro*. Moreover, XBJ can target and promote p-GSK-3β (Ser9) to reduce p-NF-κB and increase p-CREB, thereby increasing the content of anti-inflammatory factor IL-10 and decreases the levels of pro-inflammatory factor TNF-α, IL-6, IL-12 and IL-1β. Taken together, these results suggest that XBJ may protect the liver function of pyogenic ALI by targeting the promotion of GSK-3β (Ser9) to reduce cellular inflammatory factors.

## Data Availability

The original contributions presented in the study are included in the article/Supplementary Material, further inquiries can be directed to the corresponding author.

## Abbreviations

ALI, acute liver injury; ALT, alanine aminotransferase; AST, aspartate aminotransferase; CBP, CREB-binding protein; CLP, cecal ligation and perforation; CREB, cAMP-response element-binding protein protein; DAPI, 4',6-diamidino-2-phenylindole; FBS, fetal bovine serum; GSK-3β, glycogen synthase kinase 3; HE, hematoxylin and eosin; IL, interleukin; LPS, lipopolysaccharide; MODS, multiple organ dysfunction; MPO, Myeloperoxidase; MTT, 3-(4,5-dimethyl2-thiazolyl)-2,5-diphenyl-2-H-tetrazolium bromide; NF-κB, nuclear factor kappa-B; TNF-α, tumor necrosis factor-α; XBJ, xuebijing.
