# Supplementary figures and images for: Xuebijing Protects Against Septic Acute Liver Injury Based on Regulation of GSK-3β Pathway
Source: Front Pharmacol. 2021 Apr 30;12:627716. doi: 10.3389/fphar.2021.627716 (PMC8120308; doi:10.3389/fphar.2021.627716)

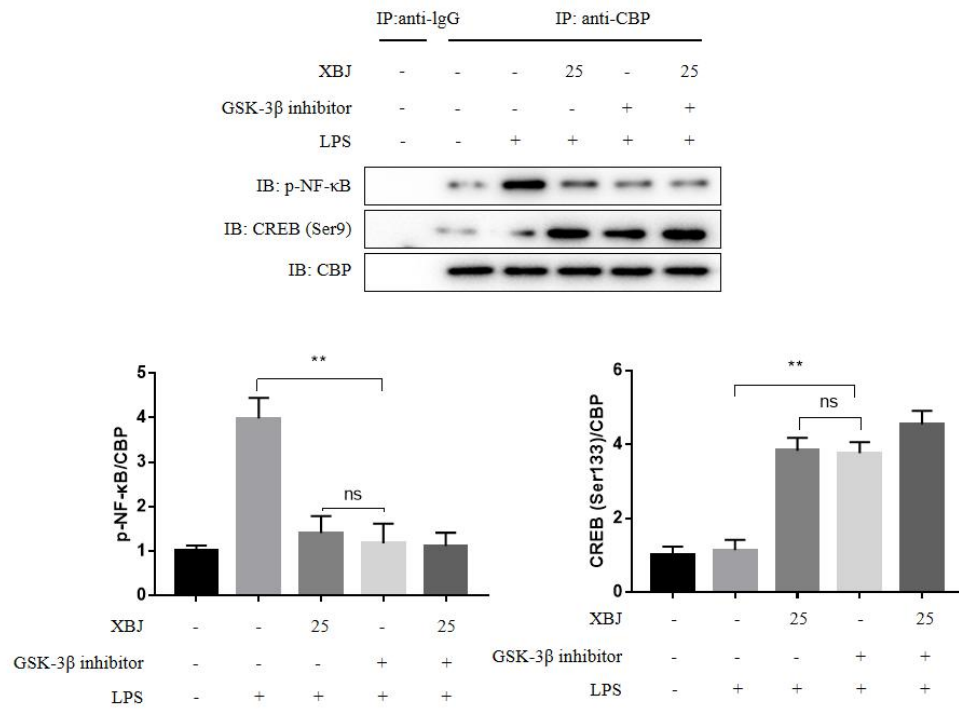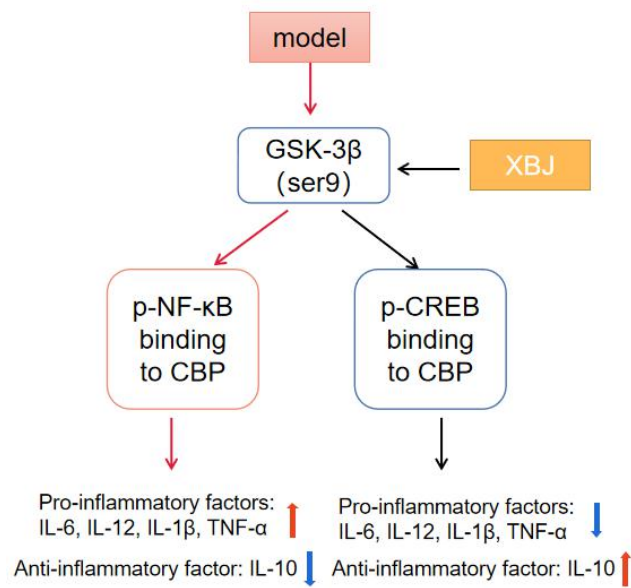

Supplement: Supplementary file 1 [file datasheet1.pdf]
